# Quantitative Computed Tomography Assessment of Bone Deficits in Ambulatory Children and Adolescents with Spina Bifida: Importance of Puberty

**DOI:** 10.1002/jbm4.10427

**Published:** 2020-11-30

**Authors:** Tishya AL Wren, Nicole M Mueske, Susan A Rethlefsen, Robert M Kay, Alexander Van Speybroeck, Wendy J Mack

**Affiliations:** ^1^ Children's Orthopaedic Center Children's Hospital Los Angeles Los Angeles CA USA; ^2^ Department of Orthopaedic Surgery University of Southern California Los Angeles CA USA; ^3^ Department of Pediatrics Children's Hospital Los Angeles and University of Southern California Los Angeles CA USA; ^4^ Department of Preventive Medicine University of Southern California Los Angeles CA USA

**Keywords:** BONE QCT, OSTEOPOROSIS, RADIOLOGY, ORTHOPEDICS, FRACTURE RISK ASSESSMENT

## Abstract

Pathologic fractures of the femur and tibia are common in youth with spina bifida (SB). These fractures may be associated with deficient bone accrual due to decreased ambulation and skeletal loading. This prospective cohort study used quantitative computed tomography (QCT) to assess three‐dimensional (3D) bone properties in children and adolescents with SB. Eighty‐three ambulatory youth with SB underwent QCT imaging of the tibia at up to four annual visits between ages 6 to 16 years (294 total visits averaging 3.5 visits/patient). A total of 177 controls without disability and 10 non‐ambulatory youth with SB underwent imaging once. Bone geometric properties (cortical bone area, cross‐sectional area, cortical thickness, cortical density, and moments of inertia) were measured at the mid‐diaphysis (50% of bone length); cross‐sectional area, cancellous density, and density‐weighted area were measured in the proximal (13% of bone length) and distal (90% of bone length) metaphyses. Bone properties were compared between the ambulatory SB and control participants, among SB neurosegmental subgroups (sacral, low lumbar, mid lumbar and above) as a function of pubertal stage (prepubertal, pubertal, postpubertal), and considering SB type (myelomeningocele, lipomyelomeningocele) using linear mixed effects models adjusted for sex, age, height percentile, and body mass index (BMI) percentile. Only cancellous density of both metaphyses and weighted area of the proximal metaphysis differed between ambulatory children with SB and controls before puberty. However, significant deficits in all bone properties manifested during and after puberty as moderate bone growth in the SB group failed to keep pace with the large increases normally observed during puberty. The bone deficits primarily affected patients with myelomeningocele, and similar deficits were observed at all neurosegmental levels except that cancellous density was closer to normal in the sacral group. Descriptive analysis of the 10 non‐ambulatory youth with SB showed greater bone deficits than ambulatory children, particularly for cancellous density in the distal metaphysis. © 2020 The Authors. *JBMR Plus* published by Wiley Periodicals LLC on behalf of American Society for Bone and Mineral Research.

## Introduction

Spina bifida (SB), including myelomeningocele (MM) and lipomyelomeningocele (LMM), is the most common permanently disabling condition in the United States^(^ ^1^ ^)^ and one of the most complex birth defects compatible with life.^(^ ^2^ ^)^ It is caused by failure of the spinal column to close completely during the first month of pregnancy. In MM, the spinal cord protrudes outside of the spinal column in a fluid‐filled sac; in LMM an abnormal fat mass connects to the spinal cord from outside the spinal column. In both cases, the spinal cord and nerves can be damaged, resulting in sensory deficits and varying degrees of paralysis and weakness in the lower extremities. The resulting functional limitations lead to decreased activity levels and ambulation,^(^ ^3^, ^4^ ^)^ reducing mechanical loading of the skeleton, which is needed to stimulate normal bone accretion. These effects are related to the level of neurologic involvement, with thoracic level patients being most affected and sacral level patients being least affected. Low bone mass and fractures are therefore common in children and adolescents with SB,^(^ ^5^ ^)^ particularly in those with higher level involvement, increasing the likelihood that they will develop osteoporosis later in life and increasing short‐term and long‐term fracture risk.

Fractures are common in children and adolescents with SB with a reported prevalence of up to 30%.^(^ ^5^ ^)^ This rate is similar to children without disability, but occurs with lower activity levels and less exposure to physical trauma. Most fractures are pathologic, occurring spontaneously or without significant trauma,^(^ ^6^, ^7^ ^)^ and occur predominantly in the lower extremity long bones.^(^ ^6^, ^7^, ^8^, ^9^, ^10^, ^11^ ^)^ These fracture patterns are atypical among children without disability, who usually fracture because of trauma and most often in the upper extremities.^(^ ^12^, ^13^ ^)^ The frequency and pattern of fractures in youth with SB suggest that fractures in this population are primarily fragility fractures associated with insufficient bone strength.

Because bone strength cannot be directly measured in vivo, imaging is often used to obtain surrogate measures of bone strength. A number of studies using dual‐energy X‐ray absorptiometry (DXA) or single‐photon absorptiometry (SPA) have examined bone mineral density (BMD) of the whole body, lumbar spine, femoral neck, distal femur, femur and tibia diaphysis, radius, and first metatarsal in patients with SB.^(^ ^14^, ^15^, ^16^, ^17^, ^18^, ^19^, ^20^, ^21^ ^)^ These studies have generally found reduced BMD in persons with SB, particularly in the lower extremities.^(^ ^22^ ^)^ BMD decreases with higher levels of neurologic involvement and lower ambulatory ability.^(^ ^14^, ^15^, ^16^, ^17^, ^19^, ^21^ ^)^ Prior fracture and periods of immobilization also reduce BMD.^(^ ^22^ ^)^ Although these studies provide insight into bone deficits in SB, they utilize two‐dimensional (2D) projection techniques (DXA and SPA) that cannot fully account for the bone's three‐dimensional (3D) structure. DXA measurements are influenced by bone and body size and inhomogeneous fat distribution,^(^ ^23^, ^24^ ^)^ which are particularly problematic for children who are growing and for patient populations with short stature and high body fat as is common in SB.^(^ ^14^, ^25^, ^26^, ^27^ ^)^

Quantitative computed tomography (QCT) is an alternative imaging technique that can capture full 3D bone structure, is minimally influenced by soft tissue, and can separately measure cortical and cancellous bone^(^ ^24^ ^)^ with relatively low dose settings. The purpose of the current study was to assess bone deficits in children and adolescents with SB using QCT. Bone density and geometry in the tibia diaphysis and metaphyses were compared between youth with SB and typically developing controls. Bone properties were also examined by SB type, neurosegmental level, and as a function of stage of pubertal development.

## Patients and Methods

This was a prospective cohort study of children with SB and controls ages 6 to 16 years. Participants with SB were recruited from local pediatric SB clinics and medical therapy units. Recruitment focused on ambulatory children with SB, but a small number of non‐ambulatory children were also included. Participants were considered ambulatory if they were at least household ambulators based on the Hoffer scale.^(^ ^28^ ^)^ Potential participants with SB were excluded if they had bilateral metal implants in the lower legs (which would interfere with imaging), currently used glucocorticoid or seizure medications, or had additional chronic conditions other than myelomeningocele and hydrocephalus. Controls were recruited from the local area through flyers and personal communication. Controls were healthy (ie, without serious medical conditions) and did not use any medications that could affect growth or development such as corticosteroids or birth control pills. Prior fracture was not an exclusion criterion in either group.

Ambulatory participants with SB underwent up to four annual visits (baseline, year 1, year 2, year 3); they were 6 to 13 years old at baseline and 9 to 16 years old at the year 3 visit. Controls and non‐ambulatory children with SB had a single visit and spanned the age range from 6 to 16 years. Baseline visits for patients with SB were conducted between December 2010 and December 2012, and follow‐up visits continued through December 2015; control data were collected from September 2010 to August 2014. A target sample size of 84 subjects per group was estimated to achieve 80% power at a significance level of 0.05 based on an effect size of 0.8 for a two‐sample *t* test comparing patients versus controls over 11 age groups assuming 10% annual attrition during longitudinal follow‐up. All study procedures were approved by the Children's Hospital Los Angeles Institutional Review Board (IRB), and written informed assent and consent were obtained from all participants and their guardians.

Demographic data including age, sex, race, and ethnicity and a brief medical history were obtained by a pediatric physician or physical therapist at the participant's first visit. Height (cm) and weight (kg) were measured at each visit by an experienced pediatric physical therapist, and body mass index (BMI, kg/m^2^) was calculated as weight/height^2^. Height was measured in a standing position for participants who could stand upright and supine for those who could not. Height, weight, and BMI percentiles for age were determined using growth charts from the Centers for Disease Control and Prevention.^(^ ^29^ ^)^ An experienced pediatric endocrinologist determined each child's Tanner stage of sexual maturity based on breast or testes development.^(^ ^30^, ^31^ ^)^ Development was classified as prepubertal (Tanner 1), pubertal (Tanner 2 to 4), or postpubertal (Tanner 5) for analysis purposes. For participants with SB, manual muscle testing was performed by the physical therapist, and functional neurosegmental level was classified based on muscle strength using the International Myelodysplasia Study Group (IMSG) criteria^(^ ^32^ ^)^ as sacral, low lumbar, or mid lumbar and above (mid lumbar+). Maturity and functional level were assessed at each visit to capture change over time.

**Fig 1:**
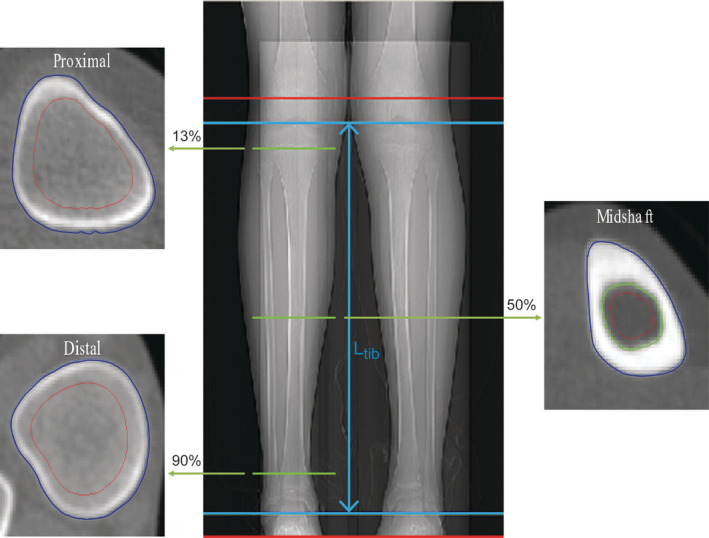
CT scan and analysis regions. Cross‐sectional area was measured as the area within the periosteum (blue contour) for all regions, and density‐weighted cross‐sectional area of the metaphyses was measured in the same region. Cortical bone area was measured as the cortical region between the periosteum and endosteum (blue and green contours), and cortical thickness, density, and moments of inertia were calculated using the same region. Cancellous density was measured within a region shrunk 30% from the blue contour and centered around the original geometric center (red contour).

Computed tomography (CT) imaging was performed to assess bone along the entire length of the tibias. All participants were assessed using the same CT scanner (Philips Gemini GXL, Philips Medical Systems Inc., Cleveland, OH, USA) and the same mineral reference phantom for simultaneous calibration (Mindways Model 3 CT Calibration Phantom; Mindways Software, Inc., Austin, TX, USA). A certified radiology technologist performed all scans. With the participant lying supine, contiguous 1‐mm slices were acquired at 90 kVp, 32 mA (100 mA for scout scan), and 1‐s rotation time along the entire length of the tibias from the knee to ankle joints with a matrix resolution of 512 × 512 pixels adjusted to the size of the participant. These scanning parameters were set much lower than standard clinical CT settings to minimize radiation exposure; the effective radiation dose was estimated to be <0.05 mSv. Each CT scan was completed in approximately 5 min. From the CT images, bone properties were measured on single slices at the mid‐diaphysis (50% bone length from the proximal surface of the intercondylar eminence to the distal surface of the medial malleolus), proximal metaphysis (13% bone length), and distal metaphysis (90% bone length) (Fig. 1). Measurements were performed using custom MATLAB (MathWorks, Natick, MA, USA) programs that analyzed contours from edge detection based on the density gradient between neighboring voxels.^(^ ^33^ ^)^ Images were not analyzed if movement artifact was observed. The primary bone outcomes measured in the midshaft were cortical bone area (CBA), cross‐sectional area (CSA, area inside the periosteum including the medullary canal), average cortical thickness, cortical bone density, and maximum (I_max_), minimum (I_min_), and polar (J) moments of inertia. In the metaphyses, the primary bone outcomes were CSA, cancellous bone density, and density‐weighted cross‐sectional area (summation of bone pixel area multiplied by pixel density producing an aggregate measure similar to total bone mass).

### Statistical analysis

Due to the small number of non‐ambulatory children with SB (*n* = 10), the primary analysis reported here compared the ambulatory participants with SB versus the controls (descriptive results are presented for the non‐ambulatory SB group). To compare bone properties between the ambulatory SB and control groups, linear mixed effects models were applied to utilize the full dataset while accommodating missing observations. First, unadjusted analyses were performed including only indicator variables for group (ambulatory SB or control), visit year (0, 1, 2, 3), and side (left or right) as fixed effects in the linear model. A random intercept term for participant was included to account for the repeated measures, modeling a random deviation of each participant from the overall group mean. Next, participant characteristics that differed between groups were considered for inclusion as covariates in adjusted analyses (see Table 1). The participant characteristics examined included age, sex, race, Tanner group, and height, weight, and BMI raw values and percentiles. Analyses stratified by sex were also performed, but are not presented because the results for both males and females were similar to the combined group with sex as a covariate.

**Table 1:** Participant Characteristics at Initial Visit

Characteristic	Control (*n* = 177)	Ambulatory SB (*n* = 83)	*p* (control versus ambulatory SB)	Non‐ambulatory SB (*n* = 10)
Age (years), mean ± SD	11.8 ± 3.1	9.7 ± 2.6	<.001	12.8 ± 2.2
Height (cm), mean ± SD	149.4 ± 17.4	130.6 ± 17.4	<.001	133.0 ± 13.4
Weight (kg), mean ± SD	47.3 ± 17.4	37.6 ± 18.5	<.001	53.5 ± 19.4
BMI (kg/m^2^), mean ± SD	20.5 ± 4.6	20.8 ± 5.7	.62	29.4 ± 7.1
Height percentile, mean ± SD	53.9 ± 27.9	27.6 ± 29.2	<.001	18.0 ± 35.1
Weight percentile, mean ± SD	62.8 ± 29.1	55.9 ± 34.7	.09	64.7 ± 35.4
BMI percentile, mean ± SD	62.8 ± 29.6	74.1 ± 27.4	.004	96.4 ± 3.9
Male, *n* (%)	95 (54%)	45 (54%)	1.00	8 (80%)
Race, *n* (%)				
White	137 (77)	77 (93)	.003	10 (100)
Black	21 (12)	3 (4)	.04	0
Asian	11 (6)	1 (1)	.11	0
Other/mixed/unknown	8 (5)	2 (2)	.51	0
Hispanic, *n* (%)	117 (66)	76 (92)	<.001	10 (100)
Tanner stage, *n* (%)			.003	
1	58 (33)	47 (57)		2 (20)
2	15 (8)	7 (8)		1 (10)
3	19 (11)	8 (10)		4 (40)
4	23 (13)	8 (10)		0 (0)
5	62 (35)	13 (16)		3 (30)
Pubertal stage, *n* (%)			<.001	
Prepubertal (Tanner 1)	58 (33)	47 (57)		2 (20)
Pubertal (Tanner 2–4)	57 (32)	23 (28)		5 (50)
Postpubertal (Tanner 5)	62 (35)	13 (16)		3 (30)
Neurosegmental level, *n* (%)				
Sacral	N/A	22 (27)	N/A	0
Low lumbar	N/A	13 (16)	N/A	0
Mid‐lumbar+	N/A	48 (58)	N/A	10 (100)
Spina bifida type, *n* (%)				
Myelomeningocele	N/A	70 (84)	N/A	10 (100)
Sacral		15 (21)		
Low lumbar		12 (17)		
Mid‐lumbar+		43 (61)		
Lipomyelomeningocele	N/A	13 (16)	N/A	0
Sacral		7 (54)		
Low lumbar		1 (8)		
Mid‐lumbar+		5 (38)		

Continuous variables are summarized as mean ± SD with group comparisons by *t* test. Categorical variables are summarized as *n* (%) with group comparisons by Fisher's exact test. Only descriptive data are presented for the non‐ambulatory SB group. Data are missing for 1 control for height, weight, and BMI raw values and percentiles. NA = not applicable.

To examine whether any differences between groups were affected by growth and development, we performed additional analyses adding the interaction between group and pubertal stage to the mixed effects models. Bone properties in ambulatory SB compared to control were further examined by neurosegmental level; in these mixed effects models, a four‐level group variable was used, with indicator variables for sacral, low lumbar, and mid‐lumbar+ relative to control. To investigate whether bone properties differed based on type of spina bifida, additional analysis was performed using a seven‐level group variable indicating spina bifida type and neurosegmental level (control, LMM sacral, LMM low lumbar, LMM mid‐lumbar+, MM sacral, MM low lumbar, MM mid‐lumbar+) in the models. All analyses were performed in Stata, version 14 (StataCorp LLC, College Station, TX, USA) with a significance level of 0.05.

## Results

The study sample included 83 ambulatory children with SB (70 MM, 13 LMM), 10 non‐ambulatory children with SB (all MM), and 177 controls. Because the ambulatory children with SB had up to four annual visits, there were 294 ambulatory SB visits (83 at baseline, 76 at year 1, 67 at year 2, 68 at year 3); there were 177 control and 10 non‐ambulatory visits because these groups completed a single visit. Missing outcome data were minimal (see Table 2 footnote) and were due to incomplete scans or metal artifact in participants with SB. No outcome measurements were missing for controls.

**Table 2:** Comparison of Bone Properties Between Ambulatory SB and Controls

Bone properties	Control (*n* = 354 person‐sides)	Ambulatory SB (*n* = 588 person‐visit‐sides)	*p*	Non‐ambulatory SB (*n* = 20 person‐sides)
Midshaft, mean ± SE				
CBA (mm^2^)	246 ± 3	215 ± 4	<.001	179 ± 15
CSA (mm^2^)	326 ± 4	290 ± 5	<.001	231 ± 19
Cortical thickness (mm)	5.2 ± 0.05	4.7 ± 0.06	<.001	4.4 ± 0.2
Cortical density (mg/cm^3^)	1,007 ± 4	1,011 ± 5	.50	1,078 ± 11
I_max_ (mm^4^)	12,263 ± 327	9,392 ± 488	<.001	5,707 ± 961
I_min_ (mm^4^)	6,396 ± 187	5,395 ± 244	.002	3,734 ± 534
J (mm^4^)	18,669 ± 542	14,782 ± 710	<.001	9,440 ± 1475
Proximal, mean ± SE				
CSA (mm^2^)	953 ± 13	942 ± 16	.62	772 ± 72
Cancellous density (mg/cm^3^)	138 ± 4	93 ± 5	<.001	70 ± 16
Weighted area (mg/cm)	2,643 ± 43	2,092 ± 55	<.001	1,552 ± 171
Distal, mean ± SE				
CSA (mm^2^)	674 ± 11	668 ± 14	.75	610 ± 70
Cancellous density (mg/cm^3^)	155 ± 3	110 ± 4	<.001	53 ± 8
Weighted area (mg/cm)	1,763 ± 29	1,497 ± 36	<.001	1,194 ± 132

Results for control and ambulatory SB groups are presented as model‐predicted mean ± SE, adjusting for sex, age, height percentile, and BMI percentile. Descriptive results for non‐ambulatory group are presented as mean ± SE. Missing data included six midshaft, eight proximal metaphysis, and 10 distal metaphysis measurements in the ambulatory SB group and one missing cortical density, proximal weighted area, and distal weighted area in the non‐ambulatory SB group.

At baseline, the ambulatory SB group was younger and less mature than the control group (Table 1), which was expected because the ambulatory SB participants did not include the oldest ages (14 to 16 years) at the initial visit due to their longitudinal follow‐up. The age was more similar between groups when all visits were included, though the control group was still slightly older (mean ± SD, 11.8 ± 3.1 versus 11.0 ± 2.8 years for controls and ambulatory SB, respectively, *p* = .005). The distribution of Tanner stage (*p* = .24) and pubertal grouping (*p* = .19) no longer differed between groups when all visits were included. The ambulatory SB group had a higher proportion of whites and a lower proportion of blacks compared with controls, along with a higher proportion of Hispanic ethnicity. The children with SB were also shorter for their age with higher BMI percentiles.

### Comparisons of ambulatory SB versus control

Adjusting for sex, age, height percentile, and BMI percentile based on the comparison of demographic and anthropometric characteristics between groups (Table 1), all geometric properties at the midshaft were significantly lower in the ambulatory SB group compared with controls (all *p* ≤ .002), but cortical density did not differ between groups (*p* = .50) (Table 2). In the metaphyses, cancellous density and density‐weighted area were significantly lower in the ambulatory SB group compared with controls (all *p* < .001), but cross‐sectional area did not differ between the two groups (*p* ≥ .62).

To examine the potential effects of race and ethnicity on the comparison of bone properties between groups, Hispanic ethnicity was included as an additional covariate in the model. Due to the small number of black participants, the potential influence of black race on the comparison of bone properties between groups was assessed through a sensitivity analysis that repeated the main analyses excluding black participants. Results were similar with or without Hispanic ethnicity in the model. Results were also similar in the sensitivity analysis excluding black participants.

### Descriptive results for non‐ambulatory SB

At the midshaft, the non‐ambulatory SB group had lower values than both the control and ambulatory SB groups for all geometric properties but had similar cortical bone density (Table 2). In the metaphyses, all bone properties were lower in the non‐ambulatory group compared with the other two groups, particularly for cancellous density in the distal metaphysis.

### Comparisons by neurosegmental level in ambulatory SB

Similar results as for the whole ambulatory SB group were obtained comparing each neurosegmental subgroup against the controls. At the midshaft, all geometric properties were lower in all neurosegmental subgroups compared with controls (all *p* ≤ .004), but cortical bone density was not significantly different (all *p* ≥ .12) (Table 3). In the metaphyses, cancellous density (all *p* < .001) and density‐weighted area (all *p* ≤ .001) were significantly lower in all neurosegmental subgroups compared with controls, but cross‐sectional area did not differ significantly from controls for any of the neurosegmental groups (*p* ≥ .18).

**Table 3:** Comparison of Bone Properties by Neurosegmental Level in Ambulatory SB

Bone properties	Control (*n* = 354 person‐sides)	Sacral (*n* = 170 person‐visit‐sides)	*p* versus control	Low lumbar (*n* = 92 person‐visit‐sides)	*p* versus control	Mid lumbar+ (*n* = 326 person‐visit‐sides)	*p* versus control
Midshaft, mean ± SE							
CBA (mm^2^)	246 ± 3	220 ± 5	<.001	212 ± 4^a^	<.001	213 ± 4	<.001
CSA (mm^2^)	365 ± 4	294 ± 6	<.001	287 ± 6	<.001	290 ± 6	<.001
Cortical thickness (mm)	5.1 ± 0.05	4.8 ± 0.07	<.001	4.6 ± 0.07^a^	<.001	4.6 ± 0.06^a^	<.001
Cortical density (mg/cm^3^)	1006 ± 4	1018 ± 6	.12	1006 ± 5^a^	.94	1009 ± 5	.67
I_max_ (mm^4^)	12,233 ± 372	9,757 ± 550	<.001	9,058 ± 523^a^	<.001	9,320 ± 508	<.001
I_min_ (mm^4^)	6,390 ± 187	5,385 ± 278	.004	5,242 ± 264	.001	5,449 ± 256	.004
J (mm^4^)	18,633 ± 542	15,129 ± 800	<.001	14,294 ± 762	<.001	14,768 ± 739	<.001
Proximal, mean ± SE							
CSA (mm^2^)	954 ± 13	927 ± 20	.27	947 ± 19	.78	949 ± 18	.82
Cancellous density (mg/cm^3^)	137 ± 3	103 ± 5	<.001	90 ± 5^a^	<.001	89 ± 5^a^	<.001
Weighted area (mg/cm)	2,637 ± 42	2,203 ± 64	<.001	2,061 ± 60^a^	<.001	2,047 ± 57^a^	<.001
Distal, mean ± SE							
CSA (mm^2^)	674 ± 11	645 ± 17	.18	666 ± 16	.70	680 ± 15^a^	.78
Cancellous density (mg/cm^3^)	154 ± 3	123 ± 5	<.001	105 ± 5^a^	<.001	104 ± 4^a^	<.001
Weighted area (mg/cm)	1,760 ± 29	1,562 ± 46	.001	1,431 ± 43^a^	<.001	1,483 ± 40	<.001

Results are presented as model‐predicted mean ± SE, adjusting for sex, age, height percentile, and BMI percentile.

^a^ *p* <.05 versus sacral.

Among the neurosegmental subgroups, the low lumbar group had lower cortical bone area, cortical thickness, cortical density, and I_max_ at the midshaft compared with the sacral group (all *p* < .03). The mid lumbar+ group also had lower cortical thickness than the sacral group (*p* = .01). Both the low lumbar and mid lumbar+ groups had lower cancellous density (all *p* < .001) and lower weighted area (*p* ≤ .07) in both metaphyses compared with the sacral group. There were no significant differences between the low lumbar and mid lumbar+ groups.

### Bone deficits in ambulatory SB as a function of pubertal stage

**Fig 2:**
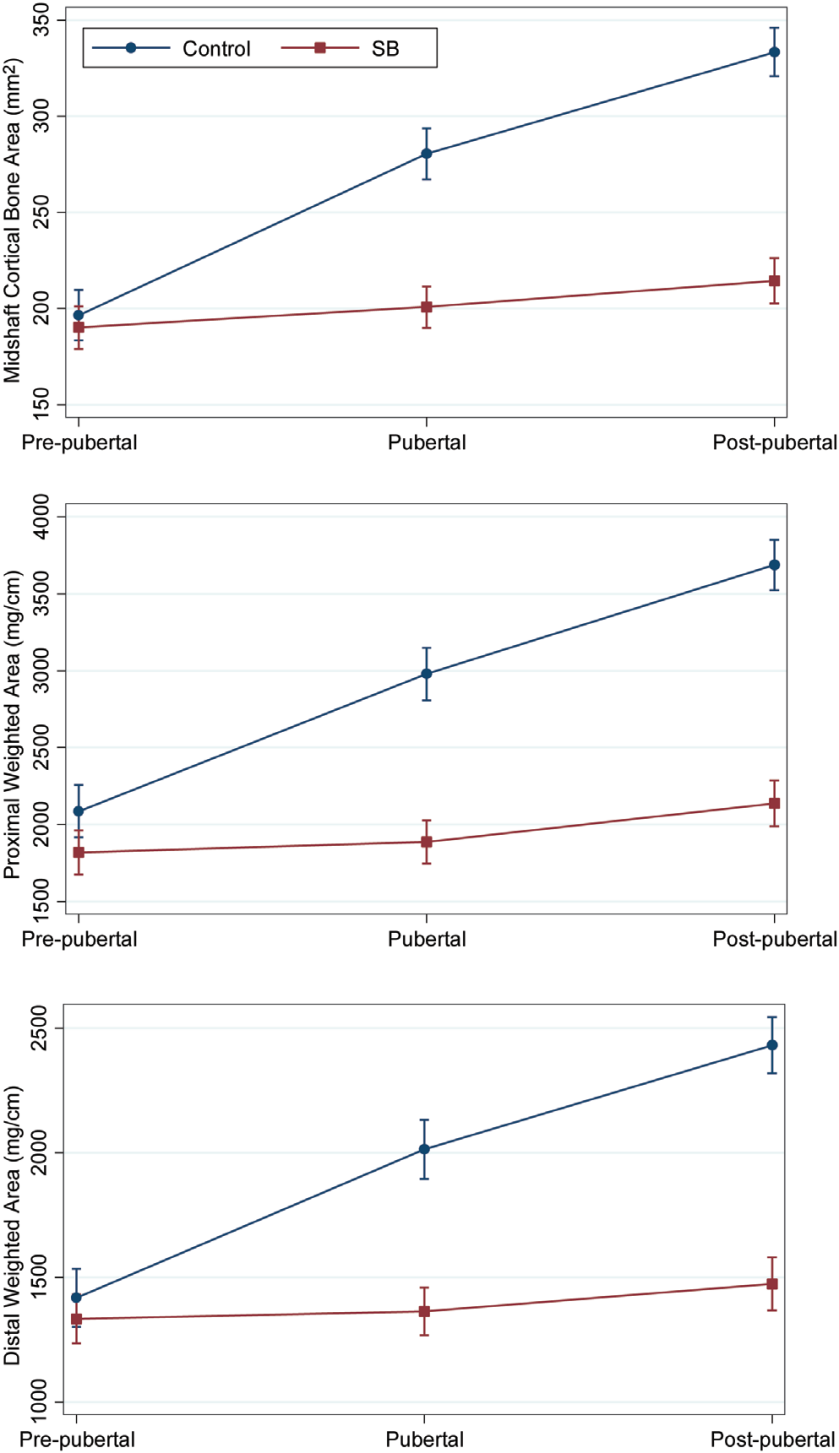
Selected bone properties (predicted mean and 95% CI) as a function of pubertal stage for ambulatory SB and control groups.

The difference in bone properties between ambulatory children with SB and controls generally increased with pubertal stage. Only cancellous density in both metaphyses (*p* < .001) and weighted area of the proximal metaphysis (*p* = .02) differed significantly between groups before puberty, but all bone properties in the diaphysis and metaphyses differed significantly during (all *p* < .03) and after (all *p* ≤ .003) puberty (Table 4). The difference between the ambulatory SB and control groups tended to increase with development as the youth with SB experienced small increases in bone properties across pubertal stages while control subjects showed much larger increases (Fig. 2). These results indicate increasing difference between the ambulatory SB and control groups as children progress through puberty.

**Table 4:** Comparison of Bone Properties Between Ambulatory SB and Controls as a Function of Pubertal Stage

	Control (*n* = 354 person‐sides)			Ambulatory SB (*n* = 588 person‐visit‐sides)			*p* control versus SB		
Bone properties	Prepubertal (*n* = 116)	Pubertal (*n* = 114)	Postpubertal (*n* = 124)	Prepubertal (*n* = 242)	Pubertal (*n* = 170)	Postpubertal (*n* = 176)	Prepubertal	Pubertal	Postpubertal
Midshaft									
CBA (mm^2^)	197 ± 7	281 ± 7	334 ± 6	190 ± 6	201 ± 6	214 ± 6	.48	<.001	<.001
CSA (mm^2^)	262 ± 9	377 ± 9	436 ± 9	259 ± 8	276 ± 7	286 ± 8	.75	<.001	<.001
Cortical thickness (mm)	4.6 ± 0.1	5.5 ± 0.1	6.2 ± 0.1	4.4 ± 0.1	4.5 ± 0.1	4.8 ± 0.1	.09	<.001	<.001
Cortical density (mg/cm^3^)	985 ± 7	1,010 ± 7	1,070 ± 6	995 ± 6	990 ± 6	1,026 ± 6	.26	.03	<.001
I_max_ (mm^4^)	6,875 ± 720	15,835 ± 724	20,803 ± 695	6,962 ± 603	8,176 ± 595	9,531 ± 624	.93	<.001	<.001
I_min_ (mm^4^)	4,055 ± 367	8,034 ± 369	10,362 ± 354	4,206 ± 307	4,857 ± 303	5,430 ± 319	.75	<.001	<.001
J (mm^4^)	10,938 ± 1065	23,890 ± 1070	31,173 ± 1027	11,173 ± 891	13,028 ± 880	14,937 ± 921	.87	<.001	<.001
Proximal									
CSA (mm^2^)	770 ± 27	1,072 ± 27	1,291 ± 26	812 ± 23	914 ± 22	969 ± 24	.24	<.001	<.001
Cancellous density (mg/cm^3^)	143 ± 6	136 ± 6	120 ± 5	96 ± 5	94 ± 5	97 ± 5	<.001	<.001	.003
Weighted area (mg/cm)	2,086 ± 87	2,978 ± 87	3,688 ± 84	1,817 ± 73	1,884 ± 72	2,137 ± 76	.02	<.001	<.001
Distal									
CSA (mm^2^)	567 ± 22	800 ± 22	843 ± 21	614 ± 18	653 ± 18	632 ± 20	.10	<.001	<.001
Cancellous density (mg/cm^3^)	146 ± 5	161 ± 5	168 ± 5	111 ± 4	106 ± 4	104 ± 5	<.001	<.001	<.001
Weighted area (mg/cm)	1,416 ± 60	2,013 ± 60	2,431 ± 58	1,333 ± 51	1,363 ± 49	1,473 ± 54	.29	<.001	<.001

Results are presented as model‐predicted mean ± SE, adjusting for sex, age, height percentile, and BMI percentile.

**Fig 3:**
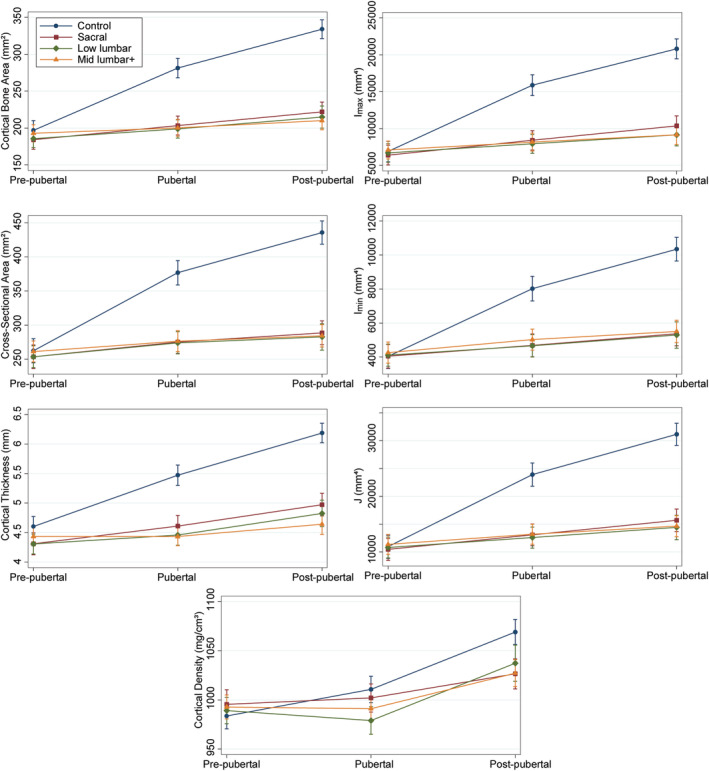
Mid‐diaphysis bone properties (predicted mean and 95% CI) as a function of pubertal stage for neurosegmental subgroups compared with controls.

**Fig 4:**
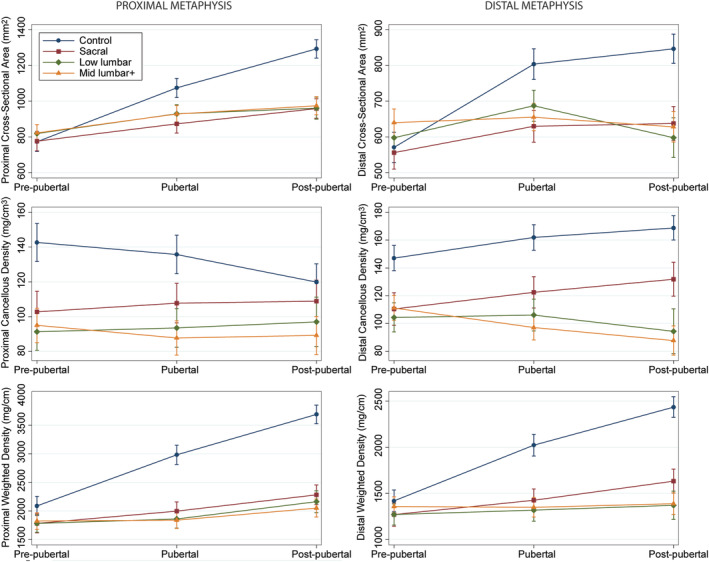
Metaphyseal bone properties (predicted mean and 95% CI) as a function of pubertal stage for neurosegmental subgroups compared with controls.

Similar patterns were observed for the neurosegmental subgroups (Table 5). Most bone properties did not differ from controls before puberty except for cancellous density in the metaphyses (all *p* < .001) and weighted area in the proximal metaphysis (*p* ≤ .02) for all neurosegmental subgroups and cortical thickness in the diaphysis for the sacral and low lumbar groups (both *p* < .03). All bone properties were lower in the neurosegmental subgroups compared with controls during and after puberty (all *p* < .05) except for cortical density during puberty (*p* = .53) and cancellous density in the proximal metaphysis postpuberty (*p* = .19) in the sacral group. Again, the magnitude of difference between the children with SB and controls increased with development as bone accrual in the youth with SB failed to keep pace with the normal rate of bone accrual observed in children without disability (Figs. 3 and 4). Similar patterns were observed in all neurosegmental groups except for cancellous density in the metaphyses which was closer to normal in the sacral compared with the low lumbar and mid lumbar+ groups.

**Table 5:** Comparison of Bone Properties Between Neurosegmental Subgroups and Controls as a Function of Pubertal Stage in Ambulatory SB

	Control (*n* = 354 person‐sides)			Sacral (*n* = 170 person‐sides)			Low lumbar (*n* = 92 person‐sides)			Mid lumbar+ (*n* = 326 person‐sides)		
Bone properties	Prepubertal (*n* = 116)	Pubertal (*n* = 114)	Postpubertal (*n* = 124)	Prepubertal (*n* = 60)	Pubertal (*n* = 44)	Postpubertal (*n* = 66)	Prepubertal (*n* = 50)	Pubertal (*n* = 32)	Postpubertal (*n* = 10)	Prepubertal (*n* = 132)	Pubertal (*n* = 94)	Postpubertal (*n* = 100)
Midshaft												
CBA (mm^2^)	197 ± 7	282 ± 7	334 ± 6	184 ± 7	**204 ± 6**	**222 ± 7**	186 ± 6	**199 ± 6**	**215 ± 8**	193 ± 6	**200 ± 6**	**210 ± 6**
CSA (mm^2^)	263 ± 9	377 ± 9	436 ± 9	254 ± 9	**276 ± 9**	**289 ± 9**	254 ± 8	**274 ± 8**	**284 ± 10**	261 ± 8	**277 ± 8**	**284 ± 8**
Cortical thickness (mm)	4.6 ± 0.1	5.5 ± 0.1	6.2 ± 0.1	**4.3 ± 0.1**	**4.6 ± 0.1**	**5.0 ± 0.1**	**4.3 ± 0.1**	**4.5 ± 0.1**	**4.8 ± 0.1**	4.4 ± 0.1	**4.4 ± 0.1**	**4.6 ± 0.1**
Cortical density (mg/cm^3^)	984 ± 7	1,009 ± 7	1,070 ± 6	996 ± 8	1,003 ± 7	**1,028 ± 8**	991 ± 7	**978 ± 7**	**1,039 ± 9**	995 ± 6	**988 ± 6**	**1,025 ± 7**
I_max_ (mm^4^)	6,898 ± 718	15,898 ± 722	20,833 ± 693	6427 ± 686	**8,435 ± 670**	**10,365 ± 699**	6,722 ± 645	**7,915 ± 656**	**9,212 ± 763**	7,112 ± 620	**8,170 ± 618**	**9,107 ± 654**
I_min_ (mm^4^)	4,054 ± 368	8,039 ± 370	10,359 ± 355	4060 ± 353	**4,696 ± 345**	**5,381 ± 360**	4,121 ± 332	**4,666 ± 338**	**5,310 ± 394**	4,278 ± 318	**5,011 ± 317**	**5,495 ± 336**
J (mm^4^)	10,960 ± 1,064	23,959 ± 1,069	31,199 ± 1,026	10,486 ± 1,011	**13,115 ± 989**	**15,712 ± 1,031**	10,846 ± 954	**12,573 ± 969**	**14,505 ± 1120**	11,397 ± 917	**13,183 ± 914**	**14,585 ± 966**
Proximal												
CSA (mm^2^)	772 ± 27	1,075 ± 27	1,293 ± 26	776 ± 27	**873 ± 27**	**959 ± 28**	819 ± 25	**929 ± 26**	**961 ± 32**	822 ± 24	**928 ± 24**	**975 ± 26**
Cancellous density (mg/cm^3^)	143 ± 6	136 ± 6	120 ± 5	**103 ± 6**	**108 ± 6**	109 ± 6	**91 ± 5**	**94 ± 6**	**97 ± 7**	**95 ± 5**	**88 ± 5**	**89 ± 6**
Weighted area (mg/cm)	2,088 ± 86	2,984 ± 86	3,692 ± 83	**1,784 ± 85**	**1,999 ± 83**	**2,284 ± 87**	**1,783 ± 79**	**1,861 ± 81**	**2,162 ± 97**	**1,823 ± 75**	**1,840 ± 75**	**2,051 ± 80**
Distal												
CSA (mm^2^)	571 ± 22	804 ± 22	847 ± 21	556 ± 23	**630 ± 23**	**638 ± 24**	598 ± 21	**687 ± 22**	**598 ± 28**	**640 ± 20**	**655 ± 19**	**628 ± 22**
Cancellous density (mg/cm^3^)	147 ± 5	162 ± 5	169 ± 4	**110 ± 6**	**122 ± 6**	**132 ± 6**	**104 ± 5**	**106 ± 6**	**94 ± 8**	**111 ± 5**	**97 ± 5**	**88 ± 5**
Weighted area (mg/cm)	1,420 ± 59	2,023 ± 60	2,436 ± 57	1,271 ± 64	**1,426 ± 62**	**1,634 ± 66**	1,271 ± 59	**1,319 ± 61**	**1,372 ± 78**	1,358 ± 54	**1,349 ± 53**	**1,389 ± 59**

Results are presented as model‐predicted mean ± SE, adjusting for sex, age, height percentile, and BMI percentile. Bold indicates significant difference from control at *p* < .05.

### Comparisons by neurosegmental level in ambulatory MM and LMM

**Fig 5:**
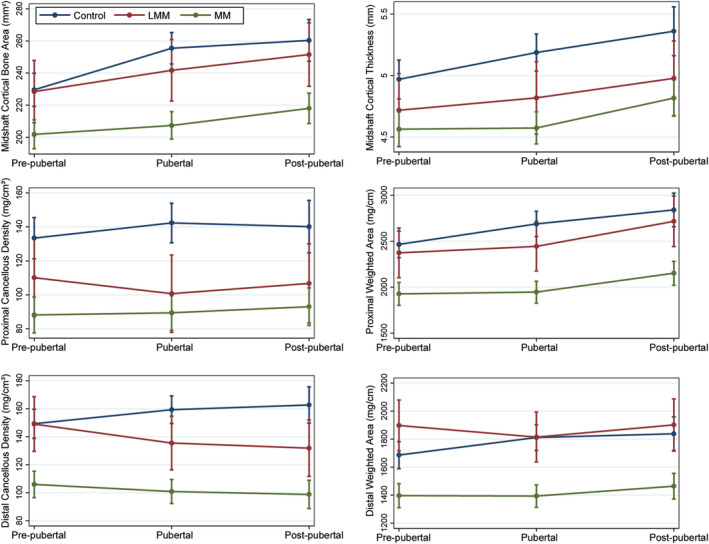
Selected bone properties (predicted mean and 95% CI) as a function of pubertal stage for lipomyelomeningocele and myelomeningocele compared with controls.

When SB type was considered, results for the ambulatory MM group were essentially the same as for the overall ambulatory SB group (results not shown). However, the ambulatory LMM group showed less difference compared with controls (Table 6, Fig. 5). At the mid‐diaphysis, only cortical thickness was significantly smaller than controls (all *p* < .05). Cortical density and the other geometric properties were similar between the LMM and control groups (*p* ≥ .17). In the metaphyses, cancellous density was lower but cross‐sectional area tended to be higher in LMM compared with controls although the differences did not always reach statistical significance. Weighted area was similar between patients with LMM and controls except for slightly lower values in the distal metaphysis at the low lumbar level (*p* = .007).

**Table 6:** Comparison of Bone Properties by Neurosegmental Level in Lipomyelomeningocele

Bone properties	Control (*n* = 354 person‐sides)	Sacral (*n* = 46 person‐visit‐sides)	*p* versus control	Low lumbar (*n* = 6 person‐visit‐sides)	*p* versus control	Mid lumbar+ (*n* = 40 person‐visit‐sides)	*p* versus control
Midshaft							
CBA (mm^2^)	246 ± 3	236 ± 10	.36	234 ± 11	.34	243 ± 10	.79
CSA (mm^2^)	326 ± 4	326 ± 12	.99	330 ± 15	.81	338 ± 12	.40
Cortical thickness (mm)	5.1 ± 0.1	4.8 ± 0.2	.047	4.7 ± 0.2	.03	4.8 ± 0.2	.046
Cortical density (mg/cm^3^)	1006 ± 4	999 ± 12	.56	1001 ± 15	.74	988 ± 13	.17
I_max_ (mm^4^)	12,251 ± 371	11,089 ± 1,259	.38	11,394 ± 1,348	.54	11,248 ± 1,263	.45
I_min_ (mm^4^)	6,402 ± 184	6,867 ± 625	.48	6,951 ± 673	.43	7,284 ± 627	.18
J (mm^4^)	18,663 ± 538	17,959 ± 1,826	.71	18,341 ± 1,955	.87	18,527 ± 1,830	.94
Proximal							
CSA (mm^2^)	956 ± 13	1062 ± 42	.02	1057 ± 48	<.001	1125 ± 43	<.001
Cancellous density (mg/cm^3^)	137 ± 3	111 ± 12	.03	101 ± 13	.007	101 ± 12	.003
Weighted area (mg/cm)	2639 ± 41	2560 ± 139	.58	2364 ± 154	.08	2440 ± 140	.17
Distal							
CSA (mm^2^)	676 ± 11	782 ± 36	.004	727 ± 42	.24	831 ± 36	<.001
Cancellous density (mg/cm^3^)	155 ± 3	142 ± 10	.21	120 ± 12	.007	141 ± 10	.18
Weighted area (mg/cm)	1760 ± 28	1882 ± 94	.22	1450 ± 112	.007	1914 ± 95	.12

Results are presented as model‐predicted mean ± SE, adjusting for sex, age, height percentile, and BMI percentile.

## Discussion

To our knowledge, this is the first large, prospective study of 3D bone structure in children and adolescents with SB. Consistent with previous studies using DXA,^(^ ^14^, ^15^, ^17^, ^20^, ^21^ ^)^ we observed lower than normal bone properties in youth with SB. Use of QCT allowed us to determine more specifically the source of these overall deficits. Deficits in the diaphysis were primarily due to geometric changes including both smaller cross‐sectional area and reduced cortical thickness, whereas deficits in the metaphyses were due to both low cancellous density and smaller bone size.

DXA is by far the most commonly used technique for assessing bone mass in SB and other patient populations due to widespread availability, low cost, and minimal radiation exposure.^(^ ^34^ ^)^ However, DXA has significant limitations when assessing bone in growing children and individuals with short stature because its 2D measurements are biased by bone size.^(^ ^34^ ^)^ In addition, DXA measurements are influenced by inhomogeneous fat distribution, particularly in individuals with high BMI.^(^ ^23^ ^)^ Lumbar spine DXA is also problematic due to scoliosis, frequent spine instrumentation, and absence of the posterior elements, which normally contribute more than half of the bone mass of the lumbar spine.^(^ ^35^ ^)^ To obtain more accurate and comprehensive bone measurements, this study utilized QCT. This was made possible by the use of low image acquisition settings designed to minimize radiation exposure at a level much lower than for clinical CT scans (effective dose <0.05 mSv). The 3D QCT images enabled us to separately examine density and geometric properties of both cortical and cancellous bone in the tibia diaphysis and metaphyses.

In more detailed analyses, we also examined the effects of neurosegmental level and pubertal development. Both age and pubertal stage were included in these analyses since youth with SB may exhibit early maturation both in terms of pubertal^(^ ^36^ ^)^ and bone^(^ ^37^ ^)^ development. These analyses revealed that the only bone deficits evident before puberty were reduced cancellous density in both metaphyses and weighted area in the proximal metaphysis (likely due to the reduced cancellous density). During puberty, typically developing children experience large increases in bone size and density, but adolescents with SB demonstrate only modest bone growth, possibly due to vitamin D deficiency, which can reduce calcium absorption and decrease bone mineralization^(^ ^38^ ^)^ and/or decreasing ambulation associated with worsening of contractures, hydrocephalus and shunt complications, increasing obesity, and the possibility of spinal cord tethering leading to development of spasticity and/or new contractures.^(^ ^39^ ^)^ The lack of bone accrual leads to greater bone deficits with advancing pubertal maturation. This may help to explain why fractures of the tibia become more common in adolescence with SB. During young childhood, most fractures in SB occur in the femur, whereas tibia fractures become more common in adolescence.^(^ ^6^, ^11^ ^)^ The femur may have a different temporal pattern of bone development, making it more susceptible to fractures earlier in life. Research specific to the femur is needed to further investigate potential differences in bone development between sites and their age‐specific fracture risk.

Examining the effects of neurosegmental level, there was surprising similarity in most bone properties amongst the neurosegmental subgroups. Multiple studies using DXA have reported lower BMD in patients with higher lesion levels and lower ambulatory ability.^(^ ^14^, ^15^, ^16^, ^17^, ^19^, ^21^, ^22^ ^)^ The most striking difference between neurosegmental subgroups observed in our study was lower cancellous density in the metaphyses in the lumbar groups compared with the sacral group. Despite this lower cancellous density, weighted area, a surrogate for total bone mass, was similar among the neurosegmental groups. Moments of inertia in the diaphysis, which serve as surrogates for long bone bending and torsional strength, were also similar among the neurosegmental subgroups. Though children with different neurosegmental involvement have different activity levels,^(^ ^3^, ^4^ ^)^ even a small amount of ambulation may be sufficient to stimulate deposition of a baseline level of bone mass. The differences observed in previous studies may be due to inclusion of non‐ambulatory participants and/or the technical inaccuracies of DXA.

The bone deficits identified in this study affected children and adolescents with MM more than LMM. Our results for participants with LMM suggest that they are not deficient in most bone properties. In the tibia diaphysis, only cortical thickness was lower than normal, and in the metaphyses only cancellous density was reduced. This was compensated for by greater than normal cross‐sectional area, resulting in weighted density being similar to controls except in the low lumbar group, which had a small sample size. Due to the smaller sample size of the LMM group, the results specific to LMM should be considered preliminary and require further investigation in larger studies focused on LMM.

The main clinical importance of bone deficits is their presumed contribution to pathologic fragility fractures. However, the relationship between bone mass and fractures has not been conclusively established specific to persons with SB. Most previous studies have been retrospective, comparing BMD between patients with or without prior fracture.^(^ ^18^, ^21^ ^)^ Establishing a relationship between bone mass and fractures would require a large prospective fracture study.

This study focused on ambulatory children with SB. Data were collected on only a small number of non‐ambulatory children to get an idea of the magnitude of difference in bone properties between ambulatory and non‐ambulatory children. Not surprisingly, the non‐ambulatory children had lower bone properties than both the control and ambulatory SB groups. Most notably, cancellous bone density was dramatically lower in the non‐ambulatory children, especially in the distal metaphysis. Bone geometric properties were only moderately lower in the non‐ambulatory group. Additional research is needed to further investigate bone deficits in non‐ambulatory children and adolescents with SB, who have weaker bones but also less exposure to loads that could lead to fracture.

An additional limitation of this study was measurement at a single slice for each analysis region, similar to the standard procedures used for peripheral QCT. The locations at 13% and 90% of bone length were selected because they fall within the metaphyses at all ages and were not obstructed by the growth plate. However, these standardized locations may not represent the same region of the metaphysis at all ages due to changes in the length and morphology of the metaphysis during growth. An alternative is to assess the full length of the metaphysis,^(^ ^40^ ^)^ which could be done from the current dataset but is more difficult to interpret. In any case, the same methodology was used for all measurements in this study, which should support the validity of the comparisons made.

This study was larger than most previous studies investigating bone in SB, both in terms of the number of participants (93 children with SB and 177 controls) and also in the number of assessments performed (294 ambulatory SB examinations). The use of mixed models in the statistical analyses enabled not only consideration of multiple visits per participant but also assessment of both limbs, resulting in 588 ambulatory SB and 354 control observations. Most previous studies examined <40 participants with SB, with the largest studies involving 60 to 80 participants,^(^ ^14^, ^19^ ^)^ This was also one of the only prospective studies of bone in SB, allowing for more controlled and standardized data collection. A minor limitation of the study was that controls were not followed longitudinally due to IRB restrictions on QCT in typically developing children intended to limit radiation exposure; they were therefore recruited with a wider baseline age range to cover the ages of the children with SB across all visits. As noted previously, the use of QCT was central to this study since QCT has distinct advantages over DXA for pediatric bone assessment.

In summary, tibial bone properties are close to normal in ambulatory children with SB before puberty except for low cancellous density in the metaphyses. However, normal pubertal growth in bone size and density is not observed in adolescents with SB, resulting in increasing bone deficits in both the diaphysis and metaphyses during and after puberty. Bone deficits affect patients with MM more than LMM and are similar across neurosegmental levels except for greater deficits in cancellous bone density in individuals with lumbar and higher level involvement. Large prospective studies are needed to relate bone deficits in SB to short‐term and long‐term fracture risk.

## Author Contributions

**Tishya Wren:** Conceptualization; data curation; formal analysis; funding acquisition; investigation; methodology; project administration; supervision; validation; visualization; writing‐original draft; writing‐review and editing. **Nicole Mueske:** Data curation; investigation; methodology; project administration; writing‐review and editing. **Susan Rethlefsen:** Investigation; methodology; writing‐review and editing. **Robert Kay:** Conceptualization; investigation; writing‐review and editing. **Alexander Van Speybroeck:** Investigation; writing‐review and editing. **Wendy Mack:** Data curation; formal analysis; software; writing‐review and editing.
